# Development of a whole-exome sequencing kit to facilitate porcine biomedical research

**DOI:** 10.1186/s13059-025-03589-4

**Published:** 2025-05-08

**Authors:** Vishwaarth Vijayakumar, Tanvi Joshi, Lobna Elkhadragy, Lawrence B. Schook, Ron C. Gaba, Mohammed El-Kebir, Kyle M. Schachtschneider

**Affiliations:** 1 Carle Illinois College of Medicine, University of Illinois at Urbana-Champaign, Urbana, IL USA; 2 Department of Animal Sciences, University of Illinois at Urbana-Champaign, Champaign, IL USA; 3 Department of Radiology, University of Illinois at Chicago, Chicago, IL USA; 4 Sus Clinicals Inc, Chicago, IL USA; 5 Department of Computer Science, University of Illinois Urbana-Champaign, Urbana, IL USA; 6 Cancer Center at Illinois, University of Illinois Urbana-Champaign, Urbana, IL USA

**Keywords:** Porcine animal models, Single nucleotide variants, Liver cancer, Exome sequencing

## Abstract

**Background:**

It is important for porcine models to replicate gene mutations present in human diseases to improve the translatability of animal studies. In this study, the high efficacy of a whole exome sequencing kit was demonstrated for the improved pig reference genome (*Sus scrofa* 11.1) to profile biomedically relevant swine breeds and enable high-depth sequencing required for intratumor heterogeneity profiling.

**Results:**

We identify a total of 751,624 single nucleotide variants (SNVs) and 113,597 insertions and deletions (INDELs) across 93 samples from 12 porcine breeds. The identified mutations and affected pathways are correlated to muscle-to-fat ratios between different porcine breeds and further inform their utility as models of obesity and cardiovascular disease. Finally, 7935 SNVs and 358 INDELs are present in an Oncopig hepatocellular carcinoma (HCC) cell line and samples from a single Oncopig HCC tumor, with pathways related to hepatic fibrosis, WNT/B-catenin, ATM signaling, and p53 signaling enriched.

**Conclusions:**

These results demonstrate the kit’s high efficacy and utility for identifying mutations in the context of obesity, cardiovascular disease, and cancer across a range of pig models used in biomedical research.

**Supplementary Information:**

The online version contains supplementary material available at 10.1186/s13059-025-03589-4.

## Background

Porcine models have proved themselves valuable for studying a wide variety of human diseases and developing therapeutic applications including but not limited to wound healing [1], cardiovascular disease [2, 3], obesity [4], organ transplantation [5], and cancer research [6]. Pigs have greater similarities to humans in terms of size, anatomy, physiology, metabolism, immunology, and genetics compared to other animal models [7]. As gene mutations implicated in human diseases such as atherosclerosis [8], coronary artery disease [9, 10], cancer [11], and obesity [12] continue to be identified, it will be important for porcine models to replicate gene mutations seen in humans and to model relevant comorbidities to ultimately improve the translatability of animal studies. Therefore, the assembly of a porcine genome sequence (*Sus scrofa*) was instrumental in accelerating porcine biomedical research [13].

Whole genome and whole exome sequencing represent two common approaches for identification of germline and somatic variation. While whole genome sequencing permits evaluation of the entire genome, whole exome sequencing is restricted to protein-coding regions. Previous studies have demonstrated that whole exome sequencing decreased costs by approximately 15-fold while allowing for evaluation of genetic variation in exon coding regions where the majority of disease-causing variants are found [14]. Previously, a whole exome sequencing kit was developed and validated for the pig reference genome *Sus scrofa* 10.2 [15]. Since then, an improved version of the pig reference genome, *Sus scrofa* 11.1 (Sscrofa11.1), was assembled that has higher continuity and accuracy by greater than 90-fold when compared to version 10.2 [16]. Therefore, there is a need for an updated porcine exome sequencing kit for utilization in conjunction with this improved pig reference genome.

The origin of each porcine breed, its history of breeding, and random mutations contribute to the genetic diversity of porcine breeds. Although a recent study identified single nucleotide variants (SNVs) to help differentiate different types of swine breeds [17], it would be helpful to investigate SNVs and insertion and deletions (INDELs) associated with porcine breeds used in biomedical research to improve modeling of human disease and translatability of results. For example, naturally occurring mutations could inform the selection of a porcine model for biomedical research to improve the translatability of studies, better understand phenotypic differences across breeds, and provide insight into mechanisms of human diseases. In addition to investigating germline genomic variation relevant to human disease research, a porcine exome sequencing kit could also be useful for delineating somatic variation in the context of porcine cancer research, as intratumor heterogeneity is increasingly being recognized as a critical component of preclinical modeling due to the impact of distinct genotypic profiles on treatment response and recurrence [18]. In the clinic setting, tumor profiling is often performed using exome or other targeted approaches. However, extensive profiling of intratumor heterogeneity in porcine cancer models is prohibitively expensive due to the use of whole genome sequencing and the high sequence depth (150 ×) required to identify somatic variation [14].

In this study, a porcine whole-exome sequencing kit based on the Sscrofa11.1 genome sequence and assembly was developed and tested to address gaps in the availability of porcine genomic tools. The utility of this kit was demonstrated across 12 domestic and minipig breeds commonly utilized in porcine biomedical research studies. Both minipig and domestic pigs were utilized to demonstrate the utility of the kit across pig breeds with significant variation in genetics and biomedically relevant phenotypes. For example, while domestic pigs typically have a full-grown weight between 140 and 300 kg, minipigs typically have a full-grown weight between 30 and 95 kg at about 2 years of age [19]. In addition, livestock animals are large and lean animals selected to build muscle mass, so while high-calorie diets in young minipigs lead to obesity, metabolic syndrome, visceral fat deposition, decreased insulin sensitivity, and increased blood cholesterol and triglycerides [20], these phenotypes are not observed in response to high-calorie diets in domestic pigs with higher muscle-to-fat ratios than minipigs [21, 22]. Furthermore, the utility of the newly developed porcine whole exome sequencing kit to characterize intratumor heterogeneity was demonstrated using the Oncopig hepatocellular carcinoma (HCC) liver cancer model [14, 23, 24]. Dysregulated pathways were investigated further to characterize the biological relevance of the model.

## Results

### Sequence and coverage statistics

The exons of protein-coding genes annotated in the Ensembl Sscrofa11.1 genome assembly have a total length of 73.84 megabases (Mb). To determine the efficacy of the developed porcine exome sequencing kit for targeting these regions, sequence data from a range of domestic and minipig breeds was generated (Table 1). Across the 12 pig breeds tested, an average of 7.38 × 10^7^ reads were obtained per sample, with an average of 7.37 × 10^7^ reads aligning to the genome. Of the reads aligning to the genome, an average of 88.92% aligned to target exon regions, resulting in an off-target rate of 11.08%. This resulted in an average coverage of 294.19 Mb per sample, 72.91 Mb of which correspond to exon sites, resulting in an average exome coverage of 98.74% at an average depth of 104.26 ×. This represents a significant enrichment compared to the average coverage of 11.08% in the off-target regions at an average depth of 11.05 ×. However, a depth greater than 5 × was only observed in 27.83% of the off-target sequenced regions. While some variability was observed between porcine breeds, these numbers were largely consistent (Table 1), demonstrating the ability of the porcine exome sequencing kit to effectively cover and enrich for Sscrofa11.1 exon regions across a range of breeds commonly utilized in biomedical research.

**Table 1:** Sequencing statistics for the whole exome sequencing kit across 12 porcine breeds

Whole exome sequencing	Total number of reads	Number of reads mapped to genome	Percent of reads aligning to exons (%)	Percent of off-target reads (%)	Average depth in off-target regions	Average depth in exon regions	Percent of exome coverage (%)
Duroc (*n* = 6)	6.75E7 (1.20E7)	6.73E7 (1.22E7)	88.24 (0.97)	11.76 (0.97)	10.77 (1.25)	96.86 (17.41)	98.81 (0.15)
Gottingen (*n* = 6)	9.28E7 (1.92E7)	9.26E7 (1.92E7)	88.83 (1.09)	11.17 (1.09)	12.12 (2.44)	135.87 (29.83)	98.89 (0.11)
Hanford (*n* = 5)	7.04E7 (6.25E6)	7.03E7 (6.24E6)	89.78 (0.63)	10.22 (0.63)	11.59 (1.03)	102.94 (9.53)	98.70 (0.09)
Large White (*n* = 6)	6.94E7 (6.69E6)	6.92E7 (6.67E6)	90.48 (0.39)	9.52 (0.39)	1.27 (0.52)	102.79 (10.04)	98.80 (0.11)
Meishan (*n* = 7)	6.62E7 (8.32E6)	6.61E7 (8.31E6)	89.01 (1.03)	10.99 (1.03)	11.12 (1.28)	96.22 (12.69)	98.68 (0.11)
Oncopig (*n* = 9)	1.13E8 (6.61E7)	1.13E8 (6.60E7)	86.00 (1.68)	13.57 (1.68)	10.28 (1.46)	131.72 (51.25)	99.92 (0.26)
Ossabaw (*n* = 9)	6.59E7 (5.07E6)	6.58E7 (5.06E6)	89.43 (0.99)	10.57 (0.99)	10.67 (1.03)	95.38 (7.55)	98.62 (0.12)
Pietrain (*n* = 5)	6.71E7 (9.36E6)	6.69E7 (9.31E6)	90.09 (0.35)	9.91 (0.35)	11.75 (1.06)	98.65 (14.06)	98.73 (0.13)
Sinclair (*n* = 7)	6.48E7 (5.87E6)	6.47E7 (5.86E6)	89.14 (0.52)	10.86 (0.52)	10.67 (0.38)	93.49 (8.56)	98.62 (0.11)
Wisconsin Miniature Swine (*n* = 19)	7.46E7 (1.14E7)	7.45E7 (1.14E7)	88.56 (0.72)	11.44 (0.72)	10.84 (0.95)	108.73 (17.02)	98.85 (0.13)
Yorkshire (*n* = 5)	5.73E7 (1.95E7)	5.72E7 (1.96E7)	89.34 (1.55)	10.66 (1.55)	10.68 (2.52)	83.49 (30.36)	98.51 (0.46)
Yucatan (*n* = 9)	6.30E7 (7.33E6)	6.29E7 (7.32E6)	89.49 (0.47)	10.51 (0.47)	10.79 (0.40)	91.11 (11.32)	98.57 (0.22)
Total (*n* = 93)	7.38E7 (2.63E7)	7.37E7 (2.63E7)	88.92 (1.34)	11.08 (1.34)	11.05 (1.33)	104.26 (25.49)	98.74 (0.21)

Mean (standard deviation)

Within each multigene family, more than 99% of all reads had a mapping quality score greater than 20, indicating less than a 1% probability that a read had been incorrectly mapped to a location within each multigene family. This provides confidence that genes within each multigene family were distinguishable from one another. Across different multigene families, an average gene depth ranging from 94.80 to 122.37 was observed compared to an average gene depth of 104.26 across all exon regions. However, variation in gene depth within each multigene family (standard deviation from 25.48 to 69.88) was observed (Additional file 1: Table S1), which may represent multiple genes that have been collapsed in the genome assembly. For example, the largest variation in average gene depth was seen in the transcription factor multigene family, varying from 20.43 (*PREB*) to 1265.13 (*FOSB*) (Additional file 1: Table S1).

### Variation across breeds

**Fig. 1:**
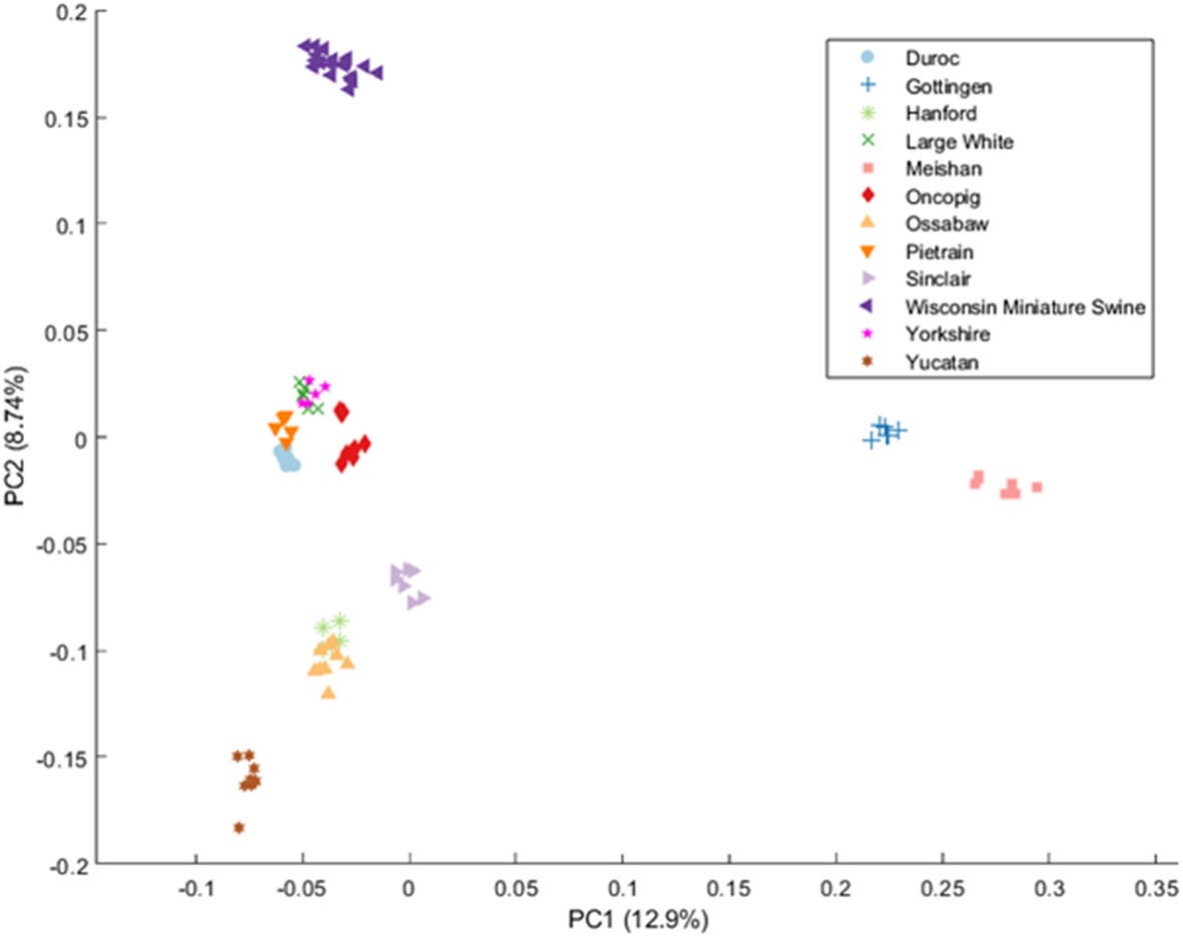
Principal component analysis of single nucleotide variants present in each porcine sample. PC, principal component

**Fig. 2:**
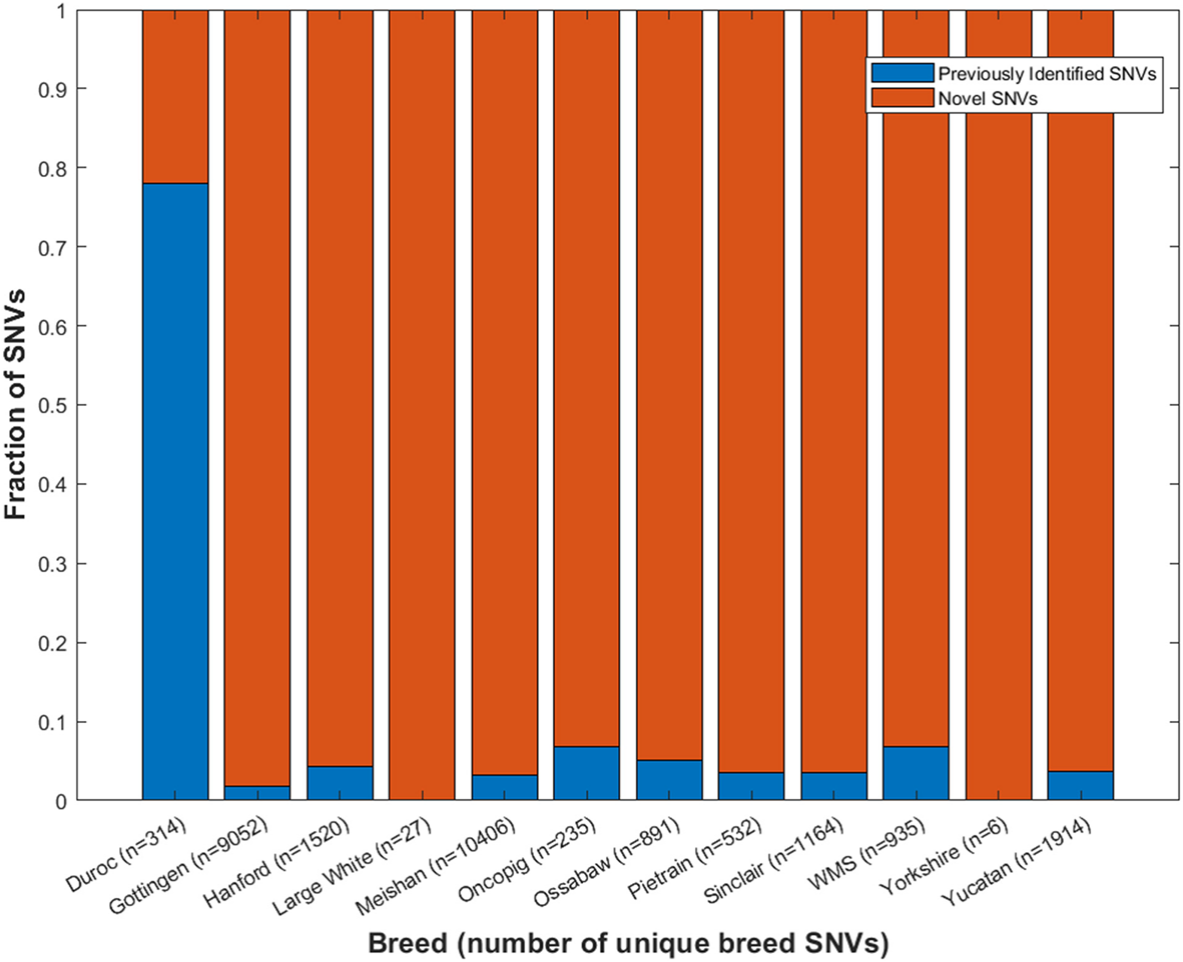
Novel vs previously identified unique breed single nucleotide variants. (SNVs = single nucleotide variants)

Principal component analysis of all SNVs resulted in breed-specific clustering consistent with previously published genetic relationships between breeds (Fig. 1) [13]. A total of 751,624 SNVs and 113,597 INDELs were observed across the 93 samples representing 12 breeds (Table 2). The number of SNVs present in each of the 12 breeds varied from 229,685 (Duroc) to 406,823 (Meishan) with an average of 309,263 SNVs. The number of INDELs present in each of the 12 breeds varied from 48,453 (Pietrain) to 65,494 (Meishan) with an average of 55,397 INDELs. Variants that were unique to 1 breed were defined as variants in > 75% of the samples representing that breed not present in other breeds, based on samples sequenced as part of this study. The number of unique breed SNVs varied from 6 (Yorkshire) to 10,406 (Meishan) with an average of 2250 SNVs. The number of unique breed INDELs present in a breed varied from 1 (Yorkshire) to 995 (Meishan) with an average of 224 INDELs. Novel unique breed SNVs and INDELs were not present in the PigVar database or in any of the 23 porcine sequencing studies in the European Variation Archive (EVA) at the time of publication. Approximately 22% of unique Duroc SNVs but > 90% of all other unique breed SNVs were novel (Fig. 2). Of all the unique breed INDELs identified in this study, only one Göttingen INDEL was previously identified. The impact of unique breed SNVs and INDELs on the protein level was quantified and stratified by high impact, moderate impact, low impact, and noncoding effects (Table 2).

**Table 2:** Number of breed-unique variant effects stratified by variant type and level of impact

	Number of samples	Total SNVs	Unique SNVs	Unique SNV effects	High effects	Moderate effects	Low effects	Noncoding effects
Duroc	6	229,685	314	1036	1	123	159	753
Gottingen	6	367,712	9052	32,369	30	2589	5151	24,599
Hanford	5	280,763	1520	5782	0	572	875	4335
Large White	6	320,205	27	84	0	2	22	60
Meishan	7	406,823	10,406	37,716	28	2565	5823	29,300
Oncopig	9	332,524	235	778	0	97	93	588
Ossabaw	9	325,796	891	3322	3	277	488	2554
Pietrain	5	259,234	532	1893	1	231	288	1373
Sinclair	7	328,441	1164	4554	9	374	723	3448
WMS	19	325,271	935	3442	12	375	591	2464
Yorkshire	5	284,382	6	13	0	2	4	7
Yucatan	9	250,322	1914	6826	4	782	972	5068
Mean		309,263	2250	8151	7	666	1266	6212
	Number of samples	Total INDELs	Unique INDELs	Unique INDEL effects	High effects	Moderate effects	Low effects	Noncoding effects
Duroc	6	48,515	76	286	14	0	2	270
Gottingen	6	61,006	904	3445	42	57	17	3329
Hanford	5	50,885	155	586	13	12	2	559
Large White	6	56,225	4	15	0	2	0	13
Meishan	7	65,494	995	3782	46	37	10	3689
Oncopig	9	59,037	23	103	1	0	2	100
Ossabaw	9	57,836	85	306	7	6	0	293
Pietrain	5	48,453	50	167	3	6	0	158
Sinclair	7	57,206	111	437	4	3	1	429
WMS	19	60,030	65	279	6	11	2	260
Yorkshire	5	51,270	1	1	0	0	0	1
Yucatan	9	48,803	216	801	11	27	3	760
Mean		55,397	224	851	12	13	3	822

*SNVs *single nucleotide variants, *INDELs* insertions and deletions, *WMS* Wisconsin Miniature Swine

### Biological implications of breed specific variation

Pathways enriched for unique breed variants were identified (Additional file 1: Tables S2–S3), including pathways associated with obesity (Table 3) and cardiovascular disease (Table 4) in several porcine breeds.

**Table 3:** Number of genes with variants associated with obesity-related pathways in various porcine breeds

Pathways	Adipogenesis	Apelin adipocyte signaling	Insulin receptor signaling	Leptin signaling in obesity	Stearate biosynthesis I	Type II diabetes mellitus signaling	White adipose tissue browning pathway
Gottingen		22 genes	37 genes	20 genes		43 genes	33 genes
Hanford				8 genes			
Meishan	38 genes		42 genes	22 genes	19 genes	43 genes	35 genes
Ossabaw				3 genes			
Sinclair	7 genes			5 genes		9 genes	
WMS				6 genes		10 genes	11 genes
Yorkshire		1 gene	1 gene	1 gene		1 gene	1 gene
Yucatan						9 genes	

*WMS* Wisconsin Miniature Swine

**Table 4:** Number of genes with variants associated with cardiac pathways in various porcine breeds

Pathways	Atherosclerosis signaling	Apelin cardiomyocyte signaling	Cardiac adrenergic signaling	Cardiac hypertrophy signaling	Cardiac hypertrophy signaling (enhanced)	Dilated cardiomyopathy signaling
Gottingen		29 genes		60 genes	111 genes	39 genes
Hanford		15 genes	15 genes		31 genes	
Meishan	33 genes			66 genes	136 genes	44 genes
Oncopig		6 genes	3 genes			
Ossabaw				6 genes		
Sinclair		6 genes		11 genes	21 genes	9 genes
Yorkshire			1 gene	1 gene		1 gene
WMS	6 genes		12 genes	12 genes		9 genes

*WMS * Wisconsin Miniature Swine

Novel high impact variants resulting in premature stop codons were identified in the following genes associated with enriched obesity pathways in Meishan pigs: *PLIN1* (adipogenesis, white adipose tissue browning), *GRB10* (insulin receptor signaling), and *ACOT4* (stearate biosynthesis I) (Table 5). A novel frameshift variant was identified in *ABCC9* (dilated cardiomyopathy signaling) in Meishan pigs. These unique Meishan variants were novel as they were not previously identified in the PigVar database or in the EVA.

**Table 5:** High impact novel unique Meishan variants associated with phenotypic differences related to obesity and cardiovascular disease

Breed	Gene	Mutation type	Amino acid length of gene	Normal gene function
Meishan	*ABCC9*	c.116 delT p.Val39fs	1603	Regulatory subunit of a cardiac ATP-sensitive potassium channel
Meishan	*ACOT4*	p.Ser6*	464	Hydrolyze fatty acyl-CoAs into fatty acids and CoA in peroxisomes and mitochondria
Meishan	*PL1 N1*	p.Ser494*	578	Mobilization of fats in adipose tissue
Meishan	*GRB10*	p.Lys534*	548	Growth factor

*Fs* frameshift

*p*.#* = indicates presence of premature stop codon at a particular amino acid

### Oncopig HCC intratumor heterogeneity

**Fig. 3:**
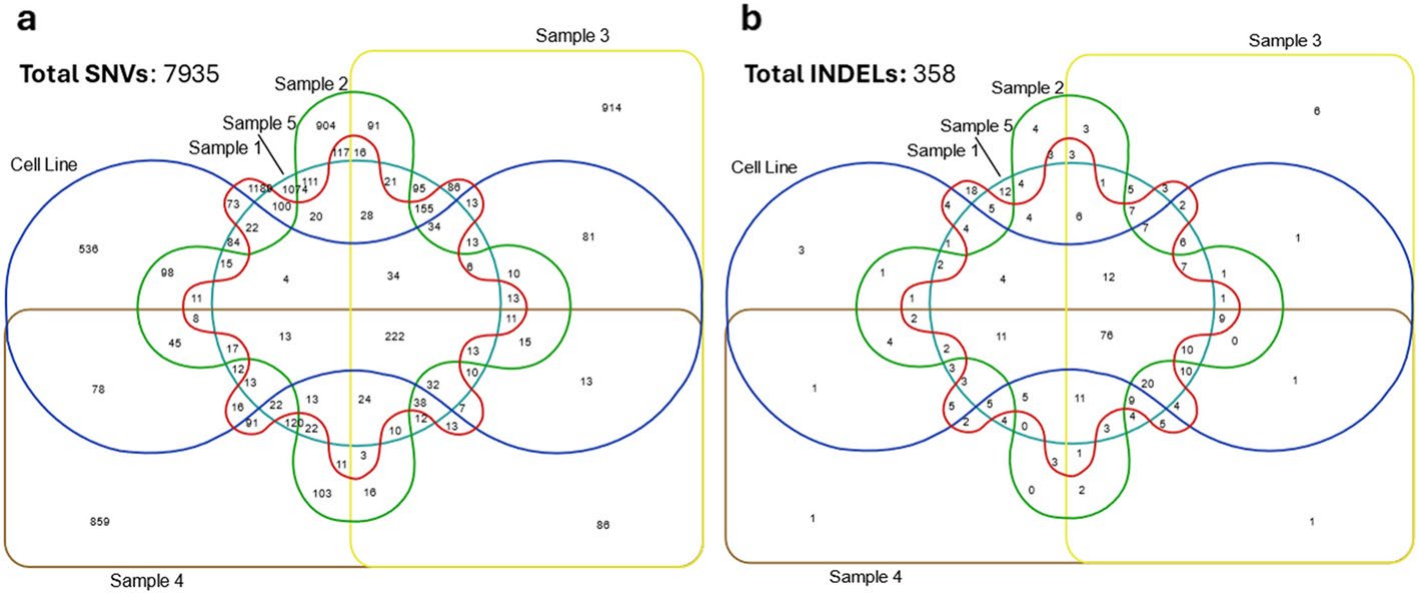
Intratumor heterogeneity depicting variants shared across Oncopig hepatocellular carcinoma model: **a** SNVs across cell line and 5 tumor regions, **b** INDELs across cell line and 5 tumor regions. SNVs, single nucleotide variants; INDELs, insertions and deletions

A total of 7935 SNVs (Additional file 1: Table S4) and 358 INDELs (Additional file 1: Table S5) were identified using whole exome sequencing within a single Oncopig HCC cell line and biopsies from 5 distinct regions of the Oncopig HCC tumor. Limited overlap of SNVs (Fig. 3a) and INDELs (Fig. 3b) was observed between regions demonstrating high intratumor heterogeneity. A total of 29,088 effects (Table 6) were associated with SNVs with 547 high impact effects, 6262 missense effects, and 530 nonsense effects present. For the 1350 effects associated with the INDELs (Table 6), 134 were predicted to be high impact with 0 missense and nonsense effects. Together, identified SNV and INDEL impacted 7958 genes (Table 6). Organ toxicity analysis identified variants in 3124 genes associated with liver hyperplasia, 869 genes associated with HCC, 222 genes associated with hepatic steatosis, and 196 genes associated with hepatic fibrosis. Of the 753 cancer driver genes from the COSMIC v101 database, 309 were affected by gene variants (Additional file 1: Table S6). Of these, 10 driver genes were affected by high impact variants (Table 7).

**Table 6:** Variant effects in Oncopig hepatocellular carcinoma model stratified by variant type and level of impact

	Number of effects associated with SNVs	Number of effects associated with INDELs	Number of total genes impacted (SNVs + INDELs)
Total	29,088	1350	7958
High impact	547	134	256
Moderate impact	6257	84	2189
Low impact	3144	8	1168
Modifier	19,140	1124	6077
Missense	6262	0	2168
Nonsense	530	0	201

*SNVs* single nucleotide variants, *INDELs* insertions and deletions

**Table 7:** High impact mutations in cancer driver genes in the Oncopig hepatocellular carcinoma model

Gene	Mutated amino acid	Number of amino acids in gene	VAF in Oncopig HCC cell line	VAF in Oncopig HCC tumor	Pathways relevant to HCC enriched for variants
CASP8	p.Glu89*	486	N/A	0.036	Molecular mechanisms of cancer MYC mediated apoptosis signaling
EZR	p.Gln322*	562	N/A	0.034	Actin cytoskeleton signaling
MAP3K1	p.Glu865*	1508	N/A	0.029	Regulation of the epithelial mesenchymal transition by growth factors Integrin signaling EGF signaling PDGF signaling
UBR5	p.Gly1240*	2705	N/A	0.036 0.010	N/A
PRPF40B	p.Ser244*	980	N/A	0.016 0.010	Spliceosomal cycle
BCL9	p.Arg667*	1426	0.010	0.047	Regulation of the epithelial-mesenchymal transition WNT/B-catenin signaling
PDGFRA	p.Trp878*	1088	N/A	0.022	Hepatic fibrosis signaling/stellate cell activation PTEN signaling PDGF signaling
SETDB1	p.Glu115*	1332	N/A	0.036	N/A
TCF7L2	p.Cys534fs	669	0.133	0.094 0.026 0.024	Hepatic fibrosis signaling Epithelial adherens junction signaling Regulation of the epithelial-mesenchymal transition WNT/B-catenin signaling
GRM3	p.Gln408*	879	N/A	0.016	N/A

*HCC* hepatocellular carcinoma, *VAF* variant allele frequency

p.#* indicates presence of premature stop codon at a particular amino acid

**Fig. 4:**
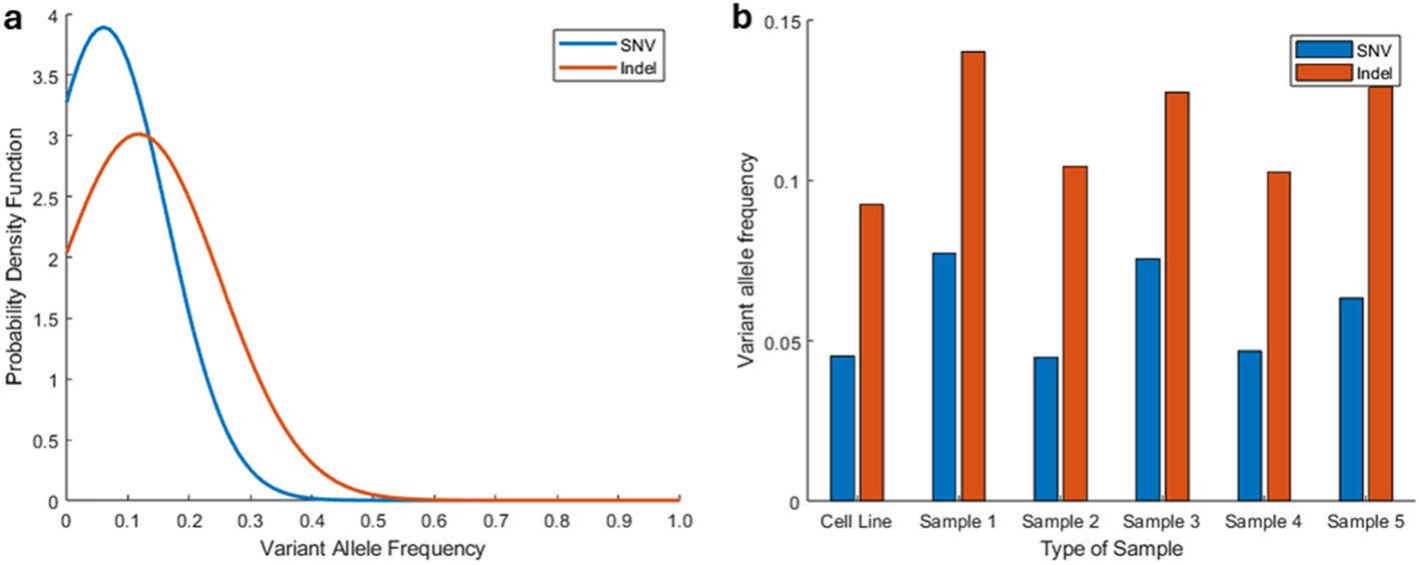
Distribution of variant allele frequencies in Oncopig HCC model: **a** Density frequency distribution of SNVs and INDELs. **b** Mean variant allele frequency across different regions. SNVs, single nucleotide variants; INDELs, insertions and deletions

The HCC cell line had a tumor purity estimate of 0.99 while the 5 tumor biopsies had tumor purity estimates between 0.18 and 0.29, which is important to note in the context of variant allele frequency (VAF) analyses. SNVs and INDELS had a mean VAF of 0.060 and 0.117, respectively (Fig. 4a) which was generally consistent across all regions of the Oncopig HCC tumor (Fig. 4b). A total of 832 SNVs (17 high impact) (Additional file 1: Table S4) and 197 INDELs (12 high impact) (Additional file 1: Table S5) identified in the HCC cell line displayed increased VAFs in at least 1 region of the in vivo Oncopig HCC tumor. Examples of genes with these high impact variants include *ATAD2* [25], *BCL9 * [26], *CP* [27]*, DBF4* [28], *DTNA* [29], *FGF23* [30], *IFIT1 * [31],* MSRB1* [32], *NID1* [33], *NSUN5* [34], *QRICH1* [35], *TP53BP1 * [36], and *ZWINT* [37] (Table 8; Additional file 1: Tables S4–S5) which have been previously linked to HCC proliferation and progression. Other genes harboring high impact variants with increased VAFs in the in vivo Oncopig HCC tumor compared to the Oncopig HCC cell line include *CCDC47*, *GFM2*, *IRF2BP1*,* LRRC10B*, *MICU3*, *MTCL1*, *OR51B2*,* PCDHAC2*, *PRUNE2*, *RDX*, *THBS3*,* URB2*, and *ZMYM6* genes (Table 8). Limited previous literature is available describing the relationship between these genes and HCC, indicating further research is required to investigate the role of these variants in HCC initiation, establishment, and growth.

**Table 8:** High impact variants with increased allele frequency in Oncopig HCC tumor compared to cell line

Gene	Variant type	VAF in Oncopig HCC cell line	Highest VAF in a region of the Oncopig HCC tumor
ATAD2	INDEL: frameshift	0.071	0.333
BCL9	SNV: stop gain	0.010	0.047
CCDC47	SNV: stop gain	0.016	0.037
CP	INDEL: frameshift	0.05	0.125
DBF4	INDEL: frameshift	0.007	0.053
DTNA	SNV: splice acceptor	0.010	0.033
ENSSSCG00000033117	SNV: stop gain	0.020	0.034
ENSSSCG00000033287	INDEL: frameshift	0.423	0.556
ENSSSCG00000042907	INDEL: frameshift	0.009	0.066
FGF23	INDEL: frameshift	0.042	0.152
GFM2	SNV: stop gain	0.013	0.042
IFIT1	INDEL: frameshift	0.019	0.083
IRF2BP1	INDEL: frameshift	0.029	0.066
LRRC10B	INDEL: frameshift	0.033	0.046
MICU3	INDEL: frameshift	0.079	0.154
MSRB1	SNV: stop gain	0.015	0.048
MTCL1	SNV: stop gain	0.009	0.016
NID1	SNV: stop gain	0.008	0.016
NSUN5	SNV: splice acceptor	0.025	0.029
OR51B2	SNV: stop gain	0.208	0.396
PCDHAC2	SNV: splice donor	0.045	1.00
PRUNE2	INDEL: frameshift	0.063	0.231
QRICH1	SNV: stop gain	0.002	0.010
RDX	INDEL: frameshift	0.031	0.109
THBS3	SNV: stop gain	0.013	0.017
TP53BP1	SNV: stop gain	0.012	0.019
URB2	SNV: stop gain	0.013	0.014
ZMYM6	SNV: stop gain	0.010	0.048
ZWINT	SNV: splice acceptor	0.010	0.032

*HCC* hepatocellular carcinoma, *VAF* variant allele frequency, *INDEL* insertion and deletion, *SNV* single nucleotide variant

Pathways enriched for genes with identified somatic variants (Additional file 1: Table S7) included molecular mechanisms of cancer (893 variant effects across 335 genes), hepatic fibrosis signaling (435 variant effects across 170 genes), *ATM* Signaling (134 variant effects across 46 genes; Additional File 2: Fig. S1), *p53* Signaling (125 variant effects across 40 genes; Additional File 2: Fig. S2), and WNT/β-catenin signaling (198 variant effects across 69 genes; Additional File 2: Fig. S3).

The molecular mechanisms of cancer pathway included variants in the following COSMIC driver genes: *CASP8* (high impact; VAF = 0.036 in HCC tumor) and *ATM* (moderate impact; VAF = 0.029 in HCC cell line and VAF = 0.012 in HCC tumor). In the *ATM* signaling pathway, a high impact mutation (premature stop codon) in *TP53BP1* (VAF = 0.012 in HCC cell line to VAF = 0.019 in HCC tumor) was identified. In the WNT/β-catenin signaling pathway, a high impact (premature stop codon) mutation (VAF = 0.010 in HCC cell line to VAF = 0.047 in HCC tumor) and missense mutation (VAF = 0.028 in HCC tumor) in *BCL9* was identified.

Somatic mutations associated with the hepatic fibrosis pathway included missense mutations in several COSMIC driver genes including *PDGFRB* (VAF = 0.020 to 0.053 in HCC tumor regions) and *PTCH1* (VAF = 0.019 in HCC tumor)*.* Finally, high-impact frameshift mutations in *TCF7L2* (VAF = 0.133 in HCC cell line and VAF = 0.024 to 0.094 in HCC tumor regions) and *CACNA1E* (VAF = 0.089 in HCC tumor) were identified in the hepatic fibrosis pathway.

## Discussion

This study aimed to develop and quantify the efficacy of the porcine exome sequencing kit to target exon regions in the updated Sscrofa11.1 genome across 12 porcine breeds. While an exome sequencing kit based on the *Sus scrofa* 10.2 reference genome was previously developed [15] that achieved 91.11% exome coverage with 67.75% of reads mapping to target regions, significant improvements in the Sscrofa 11.1 reference genome assembly and annotation warrant development of an updated kit to improve exome targeting [16]. This study demonstrated high efficacy of the developed pig exome sequencing kit to target the annotated Sscrofa11.1 exon regions with 98.74% exome coverage and an average of 88.92% of reads aligning to the target regions.

In regards to differences in the number of variants identified across breeds, it is likely that the least number of variants were identified for the Duroc breed due to the fact that the Sscrofa11.1 genome was established using a Duroc pig. Indeed, the evolutionary distance of each breed from the Duroc seems to be correlated with the number of variants identified, with domestic breeds having the least number of variants, minipigs having a moderate number of variants, and Meishan pigs having the greatest number of variants [13]. Variants identified across the 12 different domestic and minipig breeds were found to be enriched in pathways important in obesity, metabolic syndrome, and cardiac dysfunction. These results are consistent with known phenotypic differences between domestic and minipig breeds and provide insights into differential molecular alterations and pathway disruptions leading to similar disease phenotypes across breeds. For example, the leptin signaling pathway was enriched for variants in genes in 7 porcine breeds, with the highest number of genes with variants observed in the Gottingen and Meishan breeds (Table 3; Additional file 1: Table S3) commonly used to study obesity and metabolic syndrome [38–40]. In addition, both the white adipose tissue browning pathway and type II diabetes mellitus signaling pathway were enriched for the highest number of genes with variants in Gottingen, Meishan, and Wisconsin Miniature Swine. The apelin cardiomyocyte signaling pathway was enriched for the highest number of genes with variants in Gottingen and Hanford pigs (Table 4; Additional file 1: Table S3). Next, the cardiac hypertrophy signaling (Enhanced) pathway was enriched for the highest number of genes with variants in Gottingen, Hanford, and Meishan pigs (Table 4; Additional file 1: Table S3) while the atherosclerosis signaling pathway was only enriched in the Meishan breed and in the Wisconsin Miniature Swine. The dilated cardiomyopathy pathway was enriched for the highest number of genes with variants in the Gottingen and Meishan breeds. Of these breeds, the Gottingen, Hanford, and Wisconsin Miniature Swine are commonly utilized to study cardiovascular disease [41–43].

Use of our new pig exome sequencing kit in this study led to the discovery of 3 novel unique Meishan high-impact variants in the form of premature stop codons (not previously identified in the PigVar database or the EVA) in genes associated with obesity and metabolic syndrome (*PLIN1*, *GRB10*, and *ACOT4*). These variants may provide insights into molecular mechanisms driving their observed obesity phenotype. Similarly, a novel unique Meishan high-impact frameshift deletion (c.116 delT) in *ABCC9*, a regulatory subunit of a cardiac ATP-sensitive potassium channel dysregulated in the dilated cardiomyopathy pathway [44] was observed.

Together, these results demonstrate the ability of the newly developed porcine exome sequencing kit to identify high-impact variants and pathways related to human disease phenotypes such as obesity and cardiovascular disease across a range of porcine breeds commonly used in biomedical research. Future studies aimed at exploring the relationship between these high-impact variants and disease phenotypes in pigs may provide further insights into their relevance for cardiovascular disease and obesity studies.

In regard to the utility of the developed exome sequencing kit for porcine oncology studies, the high average sequencing depth in exon regions (131.72) enabled profiling intratumor heterogeneity at a substantially lower cost compared to whole genome sequencing, reducing barriers related to performing intratumor heterogeneity analyses in porcine oncology studies. Significant intratumor heterogeneity was identified in the analyzed Oncopig HCC tumor, with limited overlap of variants between different tumor regions. Pathways known to be important in HCC development and progression (molecular mechanisms of cancer, hepatic fibrosis signaling, *ATM* signaling, *p53* signaling, and WNT/B-catenin Signaling) were enriched for variants.

Genes mutated in the Oncopig HCC model were also found to be mutated in HCC patients based on comparison to the cBioportal database. For example, a high impact variant resulting in a premature stop codon (p.Glu89*) in *CASP8* was observed in the Oncopig HCC tumor. A nearly identical high impact variant (premature stop codon) was found in the clinical cBioportal database 6 amino acids downstream after accounting for homology in the proteins across species. In addition, *ATM* was found to be mutated in Oncopig HCC at a residue 11 amino acids downstream of a mutation observed in cBioPortal after accounting for homology. Finally, a premature stop codon (p.Arg1610*) was also observed in *TP53BP1*, a binding protein that helps *ATM* sense double-strand breaks. These mutations in *ATM* and *TP53BP1* could lead to the partial loss of DNA damage repair function observed in many human cancers [36], although further studies are required to confirm.

A p.Ala927 Asp mutation in *PTCH1*, which plays a role in sonic hedgehog signaling [45], was observed in Oncopig HCC 7 amino acids upstream of another Ala residue mutated in the online cBioPortal human HCC database after accounting for homology. In addition, a p.Pro925Ser mutation in *PDGFRB*, a receptor for *PDGF* correlated with alpha-fetoprotein, tumor size, and overall survival [46], was observed in the Oncopig HCC model 10 amino acids downstream (after accounting for homology) from a cBioPortal mutation that also formed a new Ser residue.

Interestingly, genes were identified with high impact variants that displayed increased VAFs in different regions of the HCC tumor compared to the HCC cell line, highlighting their potential role in in vivo HCC tumorigenesis. These potentially novel driver genes (*CCDC47*, *GFM2*, *IRF2BP1*,* LRRC10B*, *MICU3*, *MTCL1*, *OR51B2*,* PCDHAC2*, *PRUNE2*, *RDX*, *THBS3*,* URB2*, and *ZMYM6*) currently have minimal literature describing their relevance in HCC and therefore warrant further investigation. Together, these results further demonstrate the ability of the porcine whole exome sequencing kit to identify clinically relevant somatic variants and characterize intratumor heterogeneity in porcine cancer studies.

Limitations of this study include the inability to directly compare the new exome sequencing kit targeting Sscrofa11.1 with the previous version targeting Sscrofa10.2 due to the lack of publicly available data using the previous pig exome sequencing kit. Furthermore, the higher calculated performance of the developed Sscrofa 11.1 porcine exome sequencing kit may be partially due to known inaccuracies and gaps in the assembly of the *Sus scrofa* 10.2 genome [47]. In addition, when initiating the design of the exome sequencing kit, Ensembl release 93 was the most up-to-date annotation. As of publication, the current Ensembl release is release 113. The exome sequencing kit also exhibited variation in coverage in genes within a multigene family (Additional file 1: Table S1), which may represent collapsed genes in the genome assembly. This study was also limited by the relatively small number of samples present in each breed, which makes definitive statements related to the breed specificity of identified variants difficult. Indeed, increasing sample sizes could result in the identification of these variants in other breeds at a low minor allele frequency. Finally, the relationship between identified variants and observed phenotypes is mainly correlative, and the inability to confirm a causal relationship between identified mutations and phenotypes represents a limitation of this study. Future studies are required to confirm the impact of the identified variants on disease phenotypes.

Related to the applicability of the developed kit for updated and future assemblies, it is important to note that in addition to the Sscrofa 11.1 assembly for the Duroc breed, genome assemblies for 19 other breeds exist in the Ensembl database. The Sscrofa 11.1 assembly was utilized because it is the current pig reference genome most commonly used by biomedical researchers around the world. However, it is important to note that the additional genome builds currently available are highly relevant and used by researchers focused on studies involving specific breeds. When comparing the 20 genome assemblies currently available on Ensembl (Additional file 1: Table S8), relatively minor variations in the golden path length (mean = 2.48 × 10^9^ bases, standard deviation = 7.34 × 10^7^ bases), number of coding genes (mean = 2.09 × 10^4^, standard deviation = 8.41 × 10^2^), and number of gene transcripts (mean = 6.30 × 10^4^, standard deviation = 9.54 × 10^3^) were identified, suggesting this kit will be applicable for studies utilizing these genome assemblies. In order to allow researchers to evaluate the efficacy of the developed kit for other pig genome assembly and annotations, we have made the raw data from this study publicly available (PRJNA1096057 [48]; PRJEB82669 [49]). This resource will allow researchers to evaluate the utility of this kit for the specific breeds, genome assembly, and gene regions of interest to them for their particular studies before investing resources into using the developed kit for their experiments. As assemblies for additional breeds and more complete telomere-to-telomere assemblies become available, the inevitability of new genome builds replacing Sscrofa11.1 as the pig reference genome in the future, and growing interest in pangenome assemblies to better represent the genetic diversity of a given species, the long-term utility of the developed kit may be impacted. As pig pangenome assemblies become available, it will be important to continue to evaluate the utility of our kit and consider the development of additional exome sequencing kits to improve the translatability of future porcine biomedical research projects.

## Conclusions

The porcine whole exome sequencing kit developed based on the improved Sscrofa11.1 assembly and annotation results in high coverage and target specificity across a range of domestic and minipig breeds commonly used in biomedical research studies. This study also focused on breed-unique variants that had high prevalence in each porcine breed, likely increasing their level of significance. Germline mutations present in the pig breeds profiled may provide insights into the molecular mechanisms underlying disease phenotypes relevant for porcine biomedical studies. Future studies may help elucidate the relationship between identified variants and breed-specific predisposition to various diseases including cardiovascular disease, atherosclerosis, obesity, and metabolic syndrome, therefore providing further insights into their relevance as human disease models. Furthermore, the utility of the porcine whole exome sequencing kit for characterizing intratumor heterogeneity and identifying clinically relevant mutations in cancer driver genes in the Oncopig HCC model was demonstrated. Together, these results demonstrate the utility of the developed porcine whole exome sequencing kit for porcine biomedical studies utilizing a wide range of pig breeds focused on diseases with underlying germline and somatic variants.

## Methods

### Whole-exome sequencing kit development

The Ensembl [50] gene annotations for the pig from release 93, corresponding to assembly Sscrofa11.1 [16], were used for the design. The file Sus_scrofa.Sscrofa11.1.93.gtf was downloaded from the Ensembl site, all non-exon annotated regions filtered out, and the gtf file was converted to a bed file. Overlapping exon regions were merged using BEDTools v2.26 [51], resulting in a total of 217,280 exon regions spanning 73.86 Mb. The exome capture probes were purchased as the SeqCap EZ Prime Developer Probes system (Roche Nimblegen). The exome capture region was provided to Roche Nimblegen for the design of capture probes according to their standard protocols, resulting in the design of probes with an estimated 98.5% coverage of the target regions. The exome sequencing kit developed by Roche utilized in this study is available for purchase upon request from the company.

### Sample information

DNA from 93 individual pigs representing 12 porcine breeds was used for whole-exome sequencing. Breeds profiled included Duroc (*n* = 6), Gottingen Minipig (*n* = 6), Hanford (*n* = 5), Large White (*n* = 6), Meishan (*n* = 7), Oncopig (*n* = 9), Ossabaw (*n* = 9), Pietrain (*n* = 5), Sinclair (*n* = 7), Wisconsin Miniature Swine (WMS) (*n* = 19), Yorkshire (*n* = 5), and Yucatan (*n* = 9). In this study, whole exome analysis was performed using DNA from a previously sequenced (with whole genome) Oncopig HCC cell line, tumor biopsies (*n* = 5) from one Oncopig hepatocellular carcinoma tumor, and control (kidney) tissue to characterize intratumor heterogeneity [14].

### Whole exome sequencing

Whole-exome libraries were developed by the Carver High-Throughput DNA Sequencing and Genotyping Unit (HTS lab, University of Illinois, Urbana, IL) using the developed porcine whole-exome sequencing kit (4,000,036,110; Roche). Whole-exome libraries were sequenced on a NovaSeq 6000 (paired-end 150 bp reads).

### Identification of variants in porcine breeds

Raw reads were trimmed for adaptors, quality, and length using Trim_Galore v0.4.4 [52] with default parameters. Trimmed reads were aligned to the porcine reference genome (Sscrofa11.1) using BWA MEM v0.7.17 [53, 54]. BEDTools v2.26 [51] and SAMtools v1.9 [55] were used to calculate statistics related to genome and exon coverage. Multigene families were downloaded from the Molecular Signatures database for depth analyses. The GATK v4.2.6.1 pipeline for germline short variant discovery [56] was utilized to identify SNVs and INDELs via the following steps. Duplicate reads were removed using the GATK MarkDuplicates function using default parameters. The BaseRecalibrator and ApplyBQSR functions were used to adjust base quality scores using default parameters. The HaplotypeCaller function was used using default parameters to call SNVs and INDELs simultaneously and generate a GVCF file for each sample. The GenomicsDBImport function was used to consolidate GVCF files for each sample. The GenotypeGVCFs function was used to generate a set of jointly called SNVs and INDELs using default parameters. The Sscrofa 11.1 FASTA file was used as the reference sequence for this analysis. SNVs were hardfiltered using variant quality score recalibration (VQSR) with the following settings: QD < 2.0, QUAL < 30.0, SOR > 3.0, FS > 60.0, MQ < 40.0, MQRankSum < − 12.5, and ReadPosRanksum < − 8.0. INDELs were hardfiltered using VQSR with the following settings: QD < 2.0, QUAL < 30.0, FS > 200.0, and ReadPosRankSum < − 20.0. Principal component analysis (PCA) was performed using PLINK v1.9 [57] with the following parameters: –double-id, –allow-extra-chr, –set-missing-var-ids @:#, and indep-pairwise 50, 10, 0.1, with the PCA results plotted in R v4.1.0 [58]. BCFtools v1.9 [55] was used to identify variants unique to each breed that were present in > 75% of the samples for downstream analyses. Unique breed variants were also not present in a single individual from any other breed in this study. Sequencing data was downloaded from the PigVar database [59], which consists of data from 280 pigs (including a diverse cohort of Asian and European pigs) from multiple porcine sequencing studies [13, 60–63]. Sequencing data was also downloaded from 23 porcine studies from the EVA on 12/10/2024. Together, the downloaded sequencing data was utilized to identify which unique breed variants in this study were novel and which variants had been identified previously.

### Identification of porcine HCC intratumor heterogeneity

Raw reads were trimmed and aligned to the porcine reference genome (Sscrofa11.1) as described above. Duplicate reads were removed using the GATK MarkDuplicates function using default parameters [56]. Strelka v2.9 [64] was used to identify somatic SNVs and INDELs using a multi-sample workaround (https://github.com/Illumina/strelka/issues/59), keeping variants marked as PASS in at least one sample, using default parameters, and specifying –exome. SNVs were hardfiltered using VQSR with the following settings: QD < 2.0, QUAL < 30.0, SOR > 3.0, FS > 60.0, MQ < 40.0, MQRankSum < − 12.5, and ReadPosRanksum < − 8.0. INDELs were hardfiltered using VQSR with the following settings: QD < 2.0, QUAL < 30.0, FS > 200.0, and ReadPosRankSum < − 20.0. Variants that passed the filter in at least one sample were utilized for downstream analyses. Variant allele frequencies were calculated by dividing the number of alternative alleles by the total number of alleles. The PureCN v2.12.0 software [65] in R v4.4.0 was utilized to estimate tumor purity of the Oncopig HCC cell line and of biopsies from 5 locations of the Oncopig HCC tumor.

### Functional impact of variants

Functional impacts of SNVs and INDELs were predicted using SnpEff v5.0 [54] using default parameters, which categorizes the putative effect as high, medium, low, or a gene modifier. A detailed list of specific effects output by SnpEff has been previously described [54]. BioMart [50] was used to convert pig genes to their orthologous human genes (GRCh38.p13) for pathway analysis using Ingenuity Pathway Analysis [66]. Canonical pathways, upstream regulators, and organ toxicity phenotypes enriched for genes containing variants were identified with p-values < 0.05 designated as statistically significant.

### Analysis of HCC mutations

The Catalogue of Somatic Mutations in Cancer (COSMIC) v101 database of 736 causally implicated driver genes in human cancers were queried [67] to identify clinically relevant driver genes containing SNVs and INDELs. The cBioPortal online database [68, 69], which has compiled clinical genomic studies for HCC, was queried to identify mutations in the Oncopig HCC model that are clinically seen in patients. For genes with variants, BLAST software [70] on the Ensembl [50] website was utilized to determine the distance, in terms of amino acids, between mutations in the Oncopig HCC model and mutations in their orthologous genes in clinical HCC in humans.

## Supplementary Information

Additional file 1. Supplementary tables (Tables S1-S8) with results from analyses performed in this study.

Additional file 2. Supplementary figures (Figs. S1-S3) represent pathways expressed in the Oncopig hepatocellular carcinoma model.

## Data Availability

The data has been deposited under National Institutes of Health BioProject (https://www.ncbi.nlm.nih.gov/bioproject/) accession number: PRJNA1096057 [48] and in the EVA (https://www.ebi.ac.uk/eva/?Study-Browser&browserType=sgv) under the accession number: PRJEB82669 [49].
