# Supplementary material for: Development of a whole-exome sequencing kit to facilitate porcine biomedical research
Source: Genome Biol. 2025 May 8;26:118. doi: 10.1186/s13059-025-03589-4 (PMC12060350; doi:10.1186/s13059-025-03589-4)
Supplement: Supplementary file 2 — Additional file 2. Supplementary figures (Figs. S1-S3) represent pathways expressed in the Oncopig hepatocellular carcinoma model. [file 13059_2025_3589_MOESM2_ESM.docx]

**Development of a whole-exome sequencing kit to facilitate porcine biomedical research**

**Author Information**

Vishwaarth Vijayakumar^1^ ([vvijay8@illinois.edu](mailto:vvijay8@illinois.edu)), Tanvi Joshi^2^ ([tanvirj2@illinois.edu](mailto:tanvirj2@illinois.edu)), Lobna Elkhadragy^3^ ([lobna@uic.edu](mailto:lobna@uic.edu)), Lawrence B. Schook^2,4^ ([schook@illinois.edu](mailto:schook@illinois.edu)), Ron C. Gaba^3^, Mohammed El-Kebir^5,6^ ([melkebir@illinois.edu](mailto:melkebir@illinois.edu)), and Kyle M. Schachtschneider^3,4^ ([kschach2@uic.edu](mailto:kschach2@uic.edu))

^1^Carle Illinois College of Medicine, University of Illinois at Urbana-Champaign, Urbana, IL;

^2^Department of Animal Sciences, University of Illinois at Urbana-Champaign, Champaign, IL;

^3^Department of Radiology, University of Illinois at Chicago, Chicago IL

^4^Sus Clinicals Inc., Chicago, IL

^5^Department of Computer Science, University of Illinois Urbana-Champaign, Urbana, IL

^6^Cancer Center at Illinois, University of Illinois Urbana-Champaign, Urbana, IL

**Corresponding Author**

Vishwaarth Vijayakumar

Carle Illinois College of Medicine

[vvijay8@illinois.edu](mailto:vvijay8@illinois.edu)

+1-309-713-5448

**Supplementary Figures**


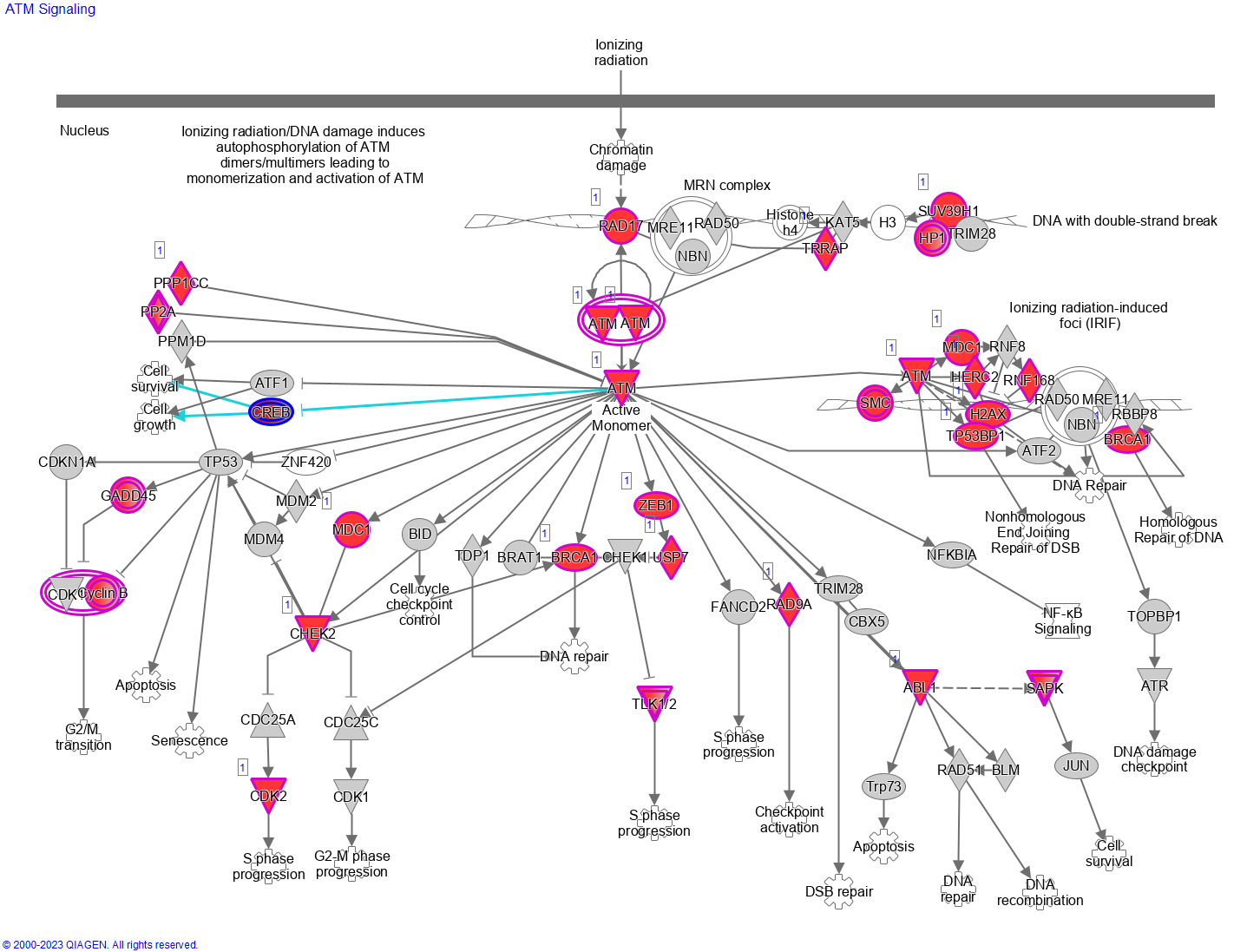
**Fig. S1.** ATM signaling pathway enriched for genes with variants present in the Oncopig
hepatocellular carcinoma model.


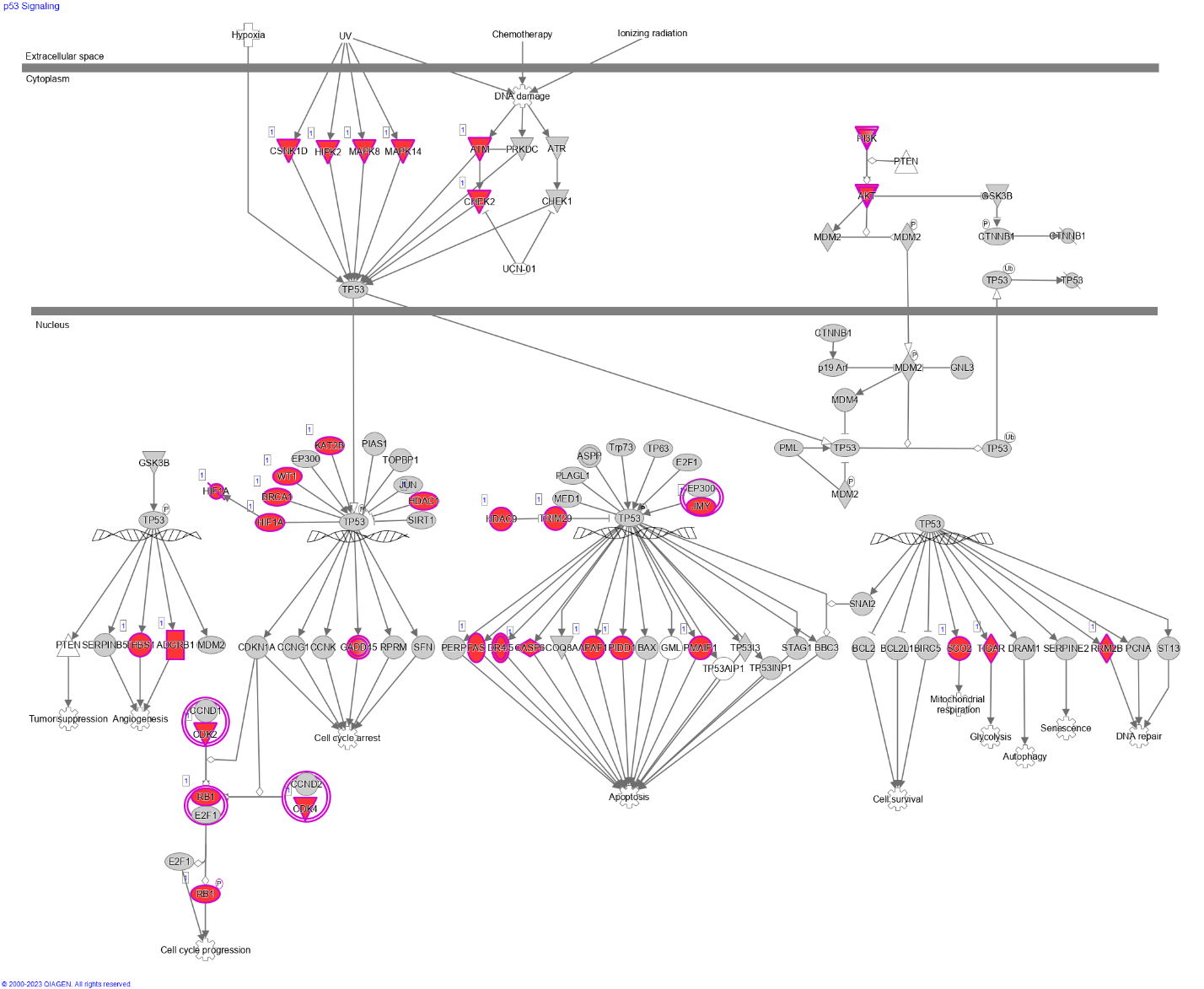


**Fig. S2.** p53 signaling pathway enriched for genes with variants present in the Oncopig hepatocellular carcinoma model.


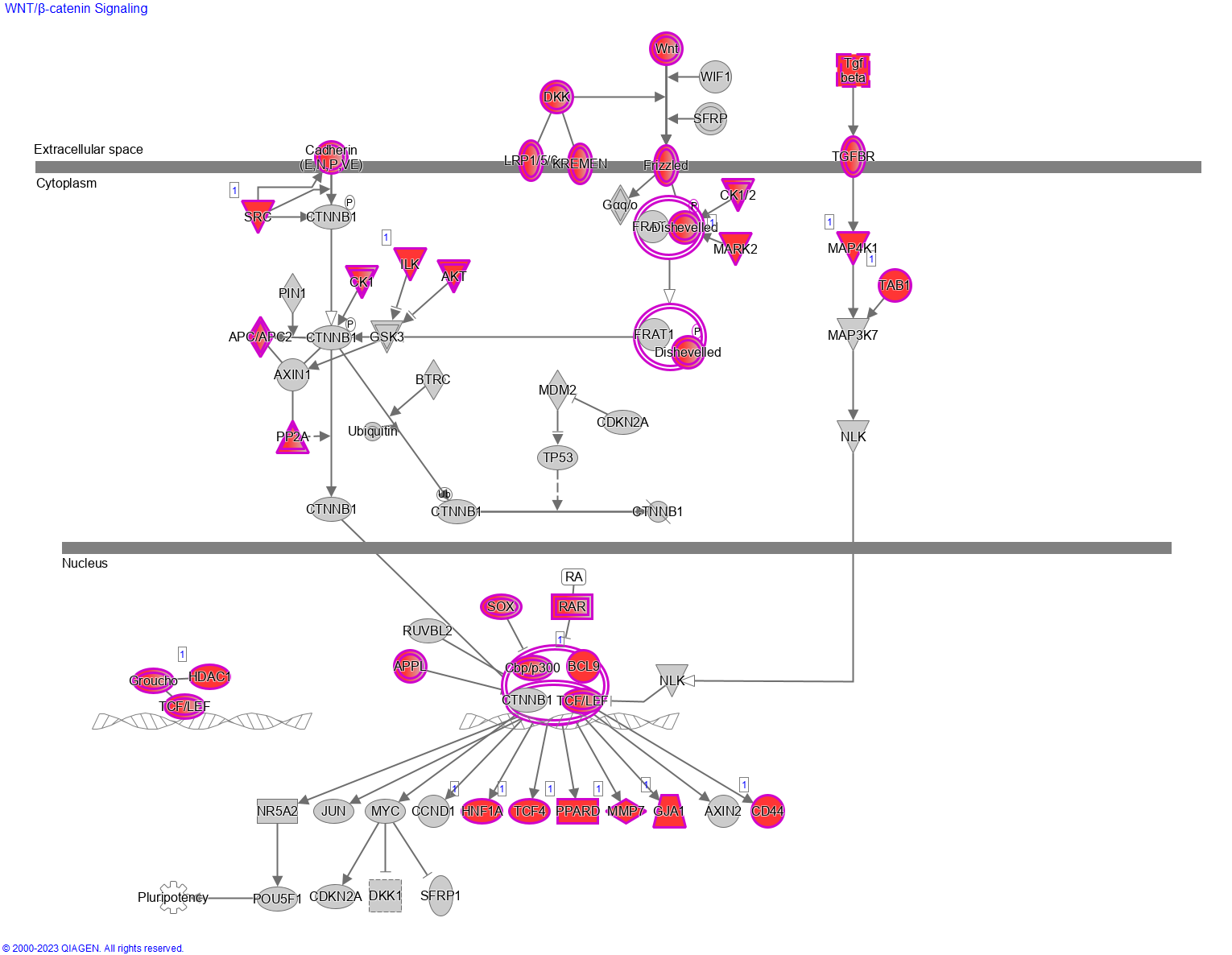


**Fig. S3.** WNT/B-catenin signaling pathway enriched for genes with variants present in the

Oncopig hepatocellular carcinoma model.
